# Applications of Amniotic Membrane and Fluid in Stem Cell Biology and Regenerative Medicine

**DOI:** 10.1155/2012/721538

**Published:** 2012-10-10

**Authors:** Kerry Rennie, Andrée Gruslin, Markus Hengstschläger, Duanqing Pei, Jinglei Cai, Toshio Nikaido, Mahmud Bani-Yaghoub

**Affiliations:** ^1^Neurogenesis and Brain Repair, National Research Council-Institute for Biological Sciences, Bldg. M-54, Ottawa, ON, Canada K1A 0R6; ^2^Department of Cellular and Molecular Medicine, Faculty of Medicine, University of Ottawa, Ottawa, ON, Canada KIH 845; ^3^Department of Obstetrics and Gynecology, Faculty of Medicine, University of Ottawa, Ottawa, ON, Canada KIH 845; ^4^Institute of Medical Genetics, Medical University of Vienna, Währinger Straße 10, 1090, Vienna, Austria; ^5^Key Laboratory of Regenerative Biology, South China Institute for Stem Cell Biology and Regenerative Medicine, Chinese Academy of Sciences, 190 Kai Yuan Avenue, Science Park, Guangzhou 510530, China; ^6^Department of Regenerative Medicine, University of Toyama Graduate School of Medicine and Pharmaceutical Sciences, 2630 Sugitani, Toyama 930-0194, Japan

## Abstract

The amniotic membrane (AM) and amniotic fluid (AF) have a long history of use in surgical and prenatal diagnostic applications, respectively. In addition, the discovery of cell populations in AM and AF which are widely accessible, nontumorigenic and capable of differentiating into a variety of cell types has stimulated a flurry of research aimed at characterizing the cells and evaluating their potential utility in regenerative medicine. While a major focus of research has been the use of amniotic membrane and fluid in tissue engineering and cell replacement, AM- and AF-derived cells may also have capabilities in protecting and stimulating the repair of injured tissues via paracrine actions, and acting as vectors for biodelivery of exogenous factors to treat injury and diseases. Much progress has been made since the discovery of AM and AF cells with stem cell characteristics nearly a decade ago, but there remain a number of problematic issues stemming from the inherent heterogeneity of these cells as well as inconsistencies in isolation and culturing methods which must be addressed to advance the field towards the development of cell-based therapies. Here, we provide an overview of the recent progress and future perspectives in the use of AM- and AF-derived cells for therapeutic applications.

## 1. Introduction

Regenerative medicine involves the use of living cells to repair, replace, or restore normal function to damaged or defective tissues and organs [1, 2]. Stem cells are viewed as promising candidates for use in cell-based therapies, owing to their capacity for self-renewal and differentiation into diverse mature progeny. However, the source of stem cells, in order to maximize the safety and efficacy of regenerative therapies, is clearly of great importance. Both adult and embryonic stem cells are commonly used to develop therapies for various preclinical models of disease and injury. Recently, induced pluripotent stem (iPS) cells, which are obtained by genetically reprogramming adult somatic cells to a pluripotent state, have also been proposed as an alternative cell source for use in regenerative medicine [3, 4]. However, a number of limitations hamper the clinical applicability of stem cells derived from either adults or developing embryos. While embryonic stem cells (ES cells) are highly proliferative and capable of differentiating into cells of all adult tissues, they pose a significant risk of tumour formation [5]. Furthermore, since ES cells are obtained by the destruction of embryos, they face serious ethical objections that have yet to be resolved. In contrast, although adult stem cells carry a reduced risk of tumorigenicity and fewer ethical restrictions, they are limited in number, have diminished differentiation capacity, and reduced proliferative potential [6, 7] which render the production of a sufficient number of cells for use in cell-based therapy difficult. Finally, despite major advances in iPS technology in recent years, reprogrammed cells often have an imperfectly cleared epigenetic memory of the source cells [8]. In addition, iPS cells are vulnerable to genomic instability [9, 10]. Due to the drawbacks associated with ES cells, adult stem cells and iPS cells, much effort has been directed at finding an alternative source of cells for use in regenerative medicine. 

Subpopulations of multipotent cells exist in both the amniotic membrane (AM) and amniotic fluid (AF). Amniotic fluid cells are obtained during amniocentesis, an important diagnostic procedure performed worldwide to evaluate the health status of the fetus during pregnancy. Amniotic epithelial (AE) and amniotic mesenchymal stromal (AMS) cells are isolated from amnion that is normally discarded following birth. These cells are therefore readily available, easily procured, and avoid the ethical issues that are associated with the use of ES cells. Subpopulations of AF- and AM-derived cells with stem cell characteristics can be maintained in the undifferentiated state in culture, but are capable of differentiating into cells representing all three germ layers under appropriate conditions [11, 12]. Unlike ES cells, AF and AE cells have not been found to form teratomas when transplanted *in vivo* [11, 13–16], and may be a safer alternative to ES cells. A comparison of AF, AE and AMS stem cells with ES cells is provided in Table 1. The use of amniotic fluid- and membrane-derived cells as cell-based therapy for a variety of indications has been extensively explored in the past decade. Here, we briefly review the findings regarding the use of AM and AF in tissue engineering and cell replacement strategies in a number of injury and disease models.

## 2. Amniotic Membrane

### 2.1. Amniotic Membrane Is a Natural Scaffold with Multiple Clinical Applications

**Figure 1:**
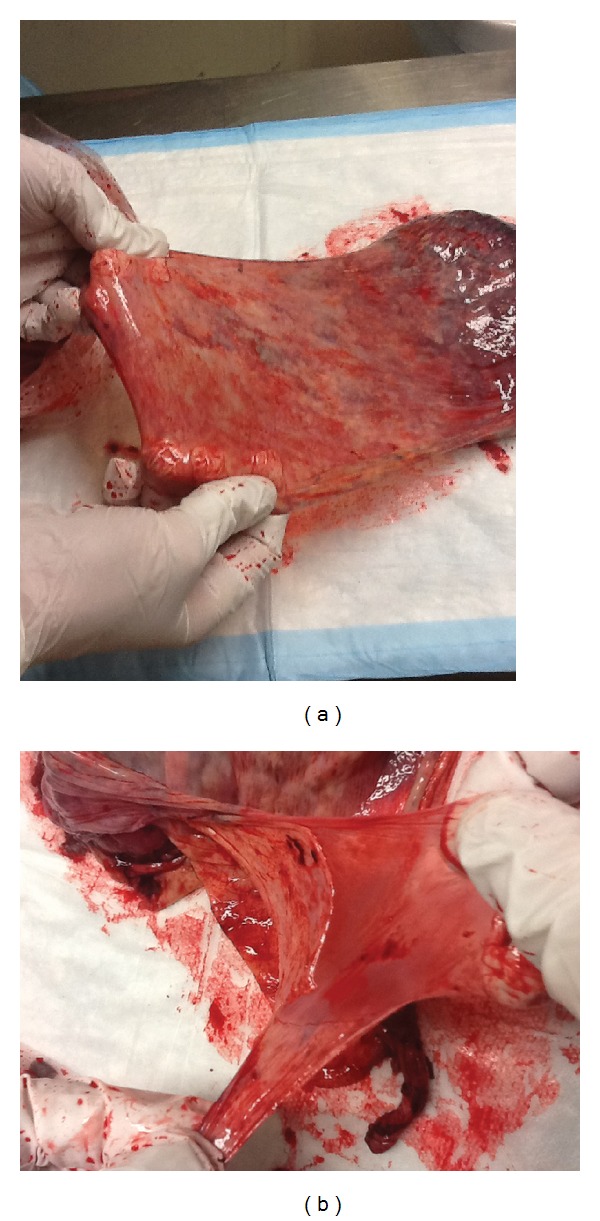
The isolation of human fetal membranes from the placenta. (a) Note the texture and elasticity of the membranes. (b) Human amniotic (left) and chorionic (right) membranes can be readily separated from each other for further purification procedures.

Human amniotic membrane (Figure 1) is the innermost fetal layer, lining the amniotic cavity and protecting the fetus during pregnancy. The outer layer, termed chorionic membrane, further separates the fetus from maternal tissues. Reports focusing on the physiological functions of fetal layers have shown that amniotic membrane not only provides a physical support for the fetus, but also serves as a metabolically active filter through a direct interaction with amniotic fluid. In particular, the transport of water and soluble materials as well as the production of growth factors, cytokines, and other bioactive molecules are regulated by amniotic membrane [17]. In addition to its role during pregnancy, amniotic membrane allows the initiation and maintenance of uterus contraction at birth [18].

The translucent, avascular, low immunogenic, anti-inflammatory, antiscarring, and wound healing properties of amniotic membrane allow this material function beyond its role *in vivo* and assume a wide range of applications in regenerative medicine [19, 20]. In fact, the clinical use of amniotic membrane has a long history, with the first reports on its application in treatment of skin burns and wounds more than a century ago [21–23]. These ground-breaking studies played a significant role in advancing the use of amniotic membrane in surgery, especially in areas such as reconstruction of the corneal and conjuctival surfaces, treatment of open ulcers and traumatic wounds, and skin transplantation [17, 20, 24, 25]. In parallel, the shelf life of amniotic membrane has been extended by irradiation, air-drying, lyophilization, cryo-preservation, and glycerol preservation techniques. These methods are expected to further expand the use of amniotic membrane in ophthalmology to treat corneal, conjunctival and limbal lesions, burns, scars and defects as well as general surgery to reconstruct skin, genitourinary tract and other surfaces [25–31]. However, the efficacy of amniotic membrane in clinical applications can only be enhanced by retaining its biological properties in the long term. This issue is especially important because the presence of key growth factors such as EGF, FGF, TGF, HGF in amniotic membranes may account for their clinical effects and mechanisms of action. Currently, a series of standardized guidelines are being developed in a number of countries to optimize the production of surgically suitable amniotic membrane from donor placenta.

### 2.2. Stem Cells in Amniotic Membrane

In addition to these strategies, various histological, biochemical, and cellular biology techniques have been used to isolate and determine the suitability of the cells in amniotic membrane for other clinical applications. Epithelial cells can be readily identified as a single layer adjacent to the amniotic fluid on one side and the basement membrane on the other side [17, 32, 33]. While epithelial cells reside on the inner layer of the amniotic membrane, mesenchymal stromal cells form the outer layer [17, 32, 33]. Both cell types have been extensively investigated for their biological properties, using a number of *in vitro* and *in vivo* models. In particular, the expression of several cellular and molecular markers has confirmed the presence of stem cells in epithelial and mesenchymal stromal cultures. Subpopulations of both AE cells and AMS cells express pluripotency markers, including OCT4, SOX2, and NANOG [13, 15, 34, 35]. AE cells express embryonic stem cell markers such as SSEA4, TRA-1-60, and TRA-1-81 [13, 36], while reports on the expression of ES cell markers by AMS cells have been inconsistent [20]. Technical issues have prevented researchers from determining whether a single human AE or AMS (hAE or hAMS) cell can differentiate into cells representative of all three germ layers after clonal expansion [37]; therefore, it remains unclear whether the human amnion harbours true pluripotent stem cells, or a mixed population of multipotent progenitor cells. Nevertheless, it is widely accepted that multiple cell types can be derived by culturing either AE or AMS cells under appropriate conditions. Several laboratories have reported neural [13, 15, 35, 38, 39], hepatic [13, 15, 40–43], cardiac [15, 34, 44], osteogenic [15, 45, 46], chondrogenic [39, 47] and adipogenic [15, 46] differentiation of both AE and AMS cells.

### 2.3. Amniotic Membrane-Derived Cells in Tissue Engineering and Cell Replacement

The development of biological substitutes to replace damaged or dysfunctional tissue may involve the construction of “replacement parts” *in vitro* for later transplantation, or the direct administration of cells to the damaged tissue [57]. AE and AMS cells have been employed for both purposes. For instance, after inducing osteogenic differentiation of human AMS cells seeded onto microcarriers, the resulting bone-like structures could be used as building blocks to form a large (2 × 1 cm) bone construct *in vitro* [58]. AE cells have been used to form tendon-like structures [59], and a double-layered skin graft (using both AE and AMS cells) capable of repairing skin defects in mice [60]. Human AE and AMS cells have also been shown to reduce liver damage in a chemically-induced model of cirrhosis [61, 62] and improve cardiac function after experimental cardiac infarction [34, 44, 63]. Furthermore, both AE and AMS cells were able to replace insulin-producing pancreatic beta cells in diabetic mice to restore normal glucose levels [64–66]. Comprehensive reviews of the differentiation potential and therapeutic use of AE and AMS cells in experimental models are available in the literature [18, 20, 37, 67–69]. 

## 3. Amniotic Fluid

### 3.1. Amniotic Fluid Is a Dynamic Environment Containing Diverse Cell Types

**Figure 2:**
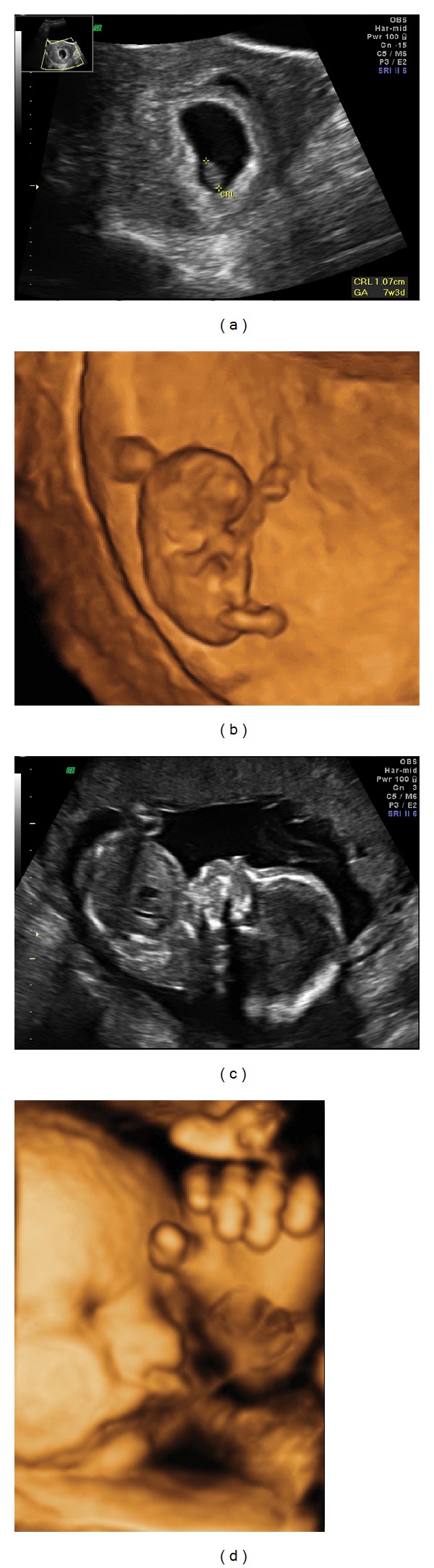
((a)-(b)) 2D (a) and 3D (b) ultrasound images of a human embryo in the first trimester. Note the relative amount of amniotic fluid compared to the size of the embryo. The fluid is mostly derived from maternal plasma at this gestational age. ((c)-(d)) A 2D ultrasound image of the fetus at 20 weeks (c) and a 3D ultrasound image of the fetal head at 36 weeks (d). Fetal urine is the main contributor to the fluid at this gestational age. Note the difference in proportion of amniotic fluid in the first (a) and second (c) trimesters.

Human amniotic fluid is a dynamic environment, which undergoes multiple developmental changes in order to sustain fetal growth and well being (Figure 2). The amniotic cavity first appears at 7-8 days after fertilization and in early gestation the amniotic fluid originates mostly from maternal plasma that crosses the fetal membranes [70]. Fetal urine first enters the amniotic space at 8–11 weeks gestation [70], and in the second half of pregnancy, fetal urine becomes the major contributor to amniotic fluid [71]. At this time, fetal skin keratinisation is complete, leading to reduced water transport across the skin and a decrease in AF osmolality. For the remainder of gestation, fluid volume is determined by different mechanisms including fetal urine production, oral, nasal, tracheal and pulmonary fluid secretion, fetal swallowing, and the contributions of the intramembranous pathway [72].

Amniotic fluid contains electrolytes, growth factors, carbohydrates, lipids, proteins, amino acids, lactate, pyruvate, enzymes, and hormones [73–76]. In addition, fluid secretions from the fetus into the AF carry a variety of fetal cells, resulting in a heterogeneous population of cells derived from fetal skin, gastrointestinal, respiratory and urinary tracts, and the amniotic membrane [77]. As the fetus develops, the volume and composition of the amniotic fluid change drastically, and the complement of cells detected in amniotic fluid samples taken at different gestational ages varies considerably [78, 79]. 

Despite this heterogeneity, cultures of amniotic fluid cells obtained by amniocentesis have been used for decades for diagnostic purposes, including standard karyotyping as well as other genetic and molecular tests. AF samples are routinely used in the evaluation of fetal lung maturity, metabolic diseases, fetal infections, and intrauterine infections. These tests have recently been complemented by applying chromosomal microarray (CMA) as a more efficient prenatal genetic screening tool to detect fetal abnormalities [80]. In this multicenter study, nearly 4400 AF samples were used to assess the performance of CMA compared with karyotyping for prenatal cytogenetic diagnosis. Interestingly, CMA analysis allowed the detection of additional genetic abnormalities in about 1 out of every 70 samples that reported a normal karyotype during routine prenatal diagnosis. These results further emphasize the importance of AF cells in providing clinically important information about the fetus. In addition, this technology can be used to routinely follow the status of different subpopulations of amniotic fluid cells in culture and identify the most suitable clones for cell-based therapies.

Generation and banking of monoclonal human AF stem cell lines with specific chromosomal aberrations or monogenic disease mutations may also help study the functional consequences of disease-causing mutations [81, 82]. As a promising approach, the use of prolonged siRNA-mediated gene silencing in AF stem cells [83] may advance our understanding of the functions of specific genes and shed light on the pathogenesis of certain naturally occurring diseases [84].

### 3.2. Stem Cells in Amniotic Fluid

The fact that amniotic fluid is commonly collected for routine diagnostic testing and is a widely accessible source of fetal cells, prompted an interest in examining the possibility that AF might contain multipotent fetal-derived cells [85]. In 2003, Prusa et al. discovered the existence of a small population of actively dividing cells in human amniotic fluid which express OCT4, a marker of pluripotent stem cells, as well as stem cell factor, vimentin and alkaline phosphatase [86]. In the same year, In 't Anker et al. reported the isolation of mesenchymal stem cells with multilineage differentiation capacity from amniotic fluid [87]. A subsequent study used immunoselection for c-kit (CD117, receptor for stem cell factor) to isolate a population of cells with high self-renewal capacity that expressed some common ES cell markers (OCT4 and SSEA4) as well as markers of somatic stem cells (CD29, CD44, CD73, CD90, and CD105) that are not typically detected in ES cells [14]. Several AF-derived clonal cell lines were established that exhibited the capability to differentiate into cell types from all three germ layers (including adipogenic, osteogenic, myogenic, endothelial, neurogenic, and hepatic cells) [14]. A number of other studies have also investigated the differentiation capacity of clonal AF-derived cells [88–93]. However, evaluation of the differentiation potential of AF-derived cells has relied heavily on expression of selected markers. Thus, further research is required to demonstrate that differentiated cells are capable of acquiring functional characteristics of the desired cell type, especially *in vivo*.

### 3.3. AF Cells in Tissue Engineering and Cell Replacement

Because they are readily accessible, pose little to no ethical concerns, and do not form teratomas *in vivo*, amniotic fluid-derived cells have been investigated as a promising alternative source of cells for use in tissue engineering and cell-based therapies. Kaviani et al. first demonstrated that mesenchymal cells from ovine or human AF could be seeded on synthetic scaffolds, as a prelude to using these cells for tissue engineering [94, 95]. Since that time, amniotic fluid-derived cells have been used in experimental settings to repair different tissues, including cartilage grafts for fetal tracheal reconstruction [96], tendons for diaphragm repair [97, 98], bone grafts [99–101], and heart valve leaflet [102–104]. *In vivo* administration of amniotic fluid-derived cells as a strategy for cell replacement has had beneficial effects in various injury models, including acute bladder injury [105], acute tubular necrosis of the kidney [106], hyperoxic lung injury [107] and ischemic heart [108]. The use of AF cells in tissue engineering and cell replacement has been extensively reviewed elsewhere [11, 12, 20] and is summarized in Table 2.

## 4. Complementary Applications of AE, AMS, and AF Cells

### 4.1. Paracrine Action of AF- and AM-Derived Cells in Tissue Repair

A common theme among several studies attempting to use AF, AE, or AMS cells for tissue repair in injury models is that, despite improving organ function, these cells often do not differentiate into the desired cell type or integrate fully into the target tissues [105, 129]. This issue may be particularly pertinent in neural applications, since the ability of AF-derived stem cells to differentiate into neurons has been a matter of debate [130, 131] and definitive evidence that AF, AE, or AMS stem cells can be induced to become mature functional neurons *in vivo* is still lacking. Nevertheless, the use of amniotic membrane- and fluid-derived cells for nervous system repair has met with some success. c-kit+ AF cells injected into injured chick embryo spinal cord increased embryo survival and reduced injury-induced haemorrhaging, although the cells failed to undergo terminal differentiation into neurons [132]. Pan et al. [113, 114] reported that AF-derived mesenchymal stromal cells improved motor function and electrophysiological indicators of nerve function in a sciatic nerve crush model, in the absence of stem cell penetration into the nerve. AF cells have also been shown to improve memory and sensory/motor functions following focal ischemia induced by middle cerebral artery occlusion (MCAO) in mice as soon as 4 days after cell injection [109]. Although the fate of the injected cells was not examined in that study, it is doubtful that AF cells could have differentiated into mature neurons capable of effectively integrating into the host circuitry to restore function on such a short time scale. Therefore, it is unlikely that cell replacement could directly account for the beneficial effects of AF cells in this study. In a rat model of Parkinson's disease, implantation of AE cells into rat striatum prevented the degeneration of nigrostriatal dopaminergic neurons, when administered prior to the neurotoxin 6-OHDA [133], and attenuated motor disturbances in rats that had previously been subjected to 6-OHDA-induced degeneration [134]. Subsequent work showed that administration of AE cells into the lateral ventricle had a similar effect, which was maintained over 10 weeks despite the fact that the majority of the transplanted cells either did not survive, or did not exhibit a dopaminergic phenotype at the end of the experiment [135]. These results further suggest that the positive effect of the transplanted AE cells was not due to their ability to replace lost nigrostriatal neurons. 

 In a number of cases, the favourable outcomes observed after AF or AM cell transplants have been attributed not to the direct replacement of lost cells, but rather to factors secreted by the cells which may serve a protective or reparative function. Such paracrine mechanisms have also been postulated to explain some of the positive effects of other stem cell types in animal models of organ/tissue injury [136–138]. Studies in which conditioned media (CM), rather than AF or AM cells themselves, have been used in injured tissues support the notion that secreted factors mediate, at least in part, the beneficial effects of the transplanted cells. For instance, AF-CM [139] and AMS-CM [140] both reinstated blood flow in a murine hindlimb ischemia model, and AF-CM increased perfusion to an ischemic skin flap [141] likely owing to the presence of proangiogenic growth factors and cytokines, including VEGF, SDF-1, and TGF-ß present within the media. AF-CM was also shown to stimulate other endogenous repair mechanisms, such as proliferation of dermal fibroblasts near the injury site in a mouse excision wound model [142] and recruitment of endothelial progenitor cells to ischemic skin flap [141]. Other paracrine mechanisms, such as the production of trophic factors [114, 143], immunomodulation [144, 145], and creation of a supportive milieu for regeneration [146] might also contribute to the ability of AF- or AM-derived cells to limit damage and/or stimulate repair of injured tissue. 

### 4.2. AF- and AM-Derived Cells for Delivery of Beneficial Factors

Although AF- and AM-derived cells appear to have natural protective and reparative functions, they may also be used for efficient biodelivery of specific factors to enhance the protection or repair of damaged tissue through genetic modification. Accordingly, it was recently reported that AF mesenchymal stromal cells engineered to express elevated levels of GDNF ameliorated motor deficits in rats subjected to sciatic nerve crush, beyond the improvement observed with green fluorescent protein (GFP)-transduced cells [147]. To extend this research to CNS applications, we are currently assessing the neuroprotective capacity of GDNF-expressing AF cells in a mouse motor cortex injury model (unpublished data). Both AE [148] and AMS [149] cells have also been used to deliver neurotrophic factors (GDNF and BDNF, resp.) to ischemic rat brain, and in both cases, enhanced recovery using GDNF- or BDNF-expressing cells was observed, relative to GFP-expressing cells. 

AF- and AM-derived cells might be suitable for delivery of diverse compounds for a variety of diseases. For instance, a handful of recent studies have made use of AF cells for biodelivery of anticancer therapeutics. Yin et al. engineered AF mesenchymal stromal cells to express the antiangiogenic factor endostatin and the prodrug-activating enzyme secretable carboxylesterase 2 (sCE2) to treat glioma. sCE2 converts the antitumour drug CPT11 into its active form. By injecting the engineered cells along with glioma-forming cells prior to treatment with CPT11, the AF cells boosted the conversion of the prodrug to its active form selectively at the tumour site, inhibiting proliferation, increasing apoptosis, and decreasing the population of glioma stem cells [150]. Similarly, expression in AF cells of cytosine deaminase and thymidine kinase, which act as suicide genes by converting two cancer prodrugs to their active toxic forms, inhibited the growth of breast tumours in a xenograft mouse model and prevented both the damage to the surrounding tissue and the physical side effects that were observed when the active drugs were directly administered [151]. These studies highlight a potential role for AF cells in biodelivery of a wide range of compounds. 

 Presumably, all of the above-mentioned studies have relied on bulk release of secreted factors into the extracellular space to mediate the beneficial effects of AF or AM cells. However, we are also investigating the possibility that AF cells could be used for direct cellular delivery of certain types of molecules via gap junctional communication, as has been suggested by Brink et al. [152] for bone marrow mesenchymal stromal cells. AF cells express connexins, the proteins that make up gap junction hemichannels, and are capable of establishing gap junctional communication with cultured cortical cells, as evidenced by dye transfer [112]. Given the induction of connexin expression surrounding a surgical lesion to motor cortex, [112] as well as in other models of brain injury [153–155], it is hoped that AF cells might be capable of delivering small molecules through gap junctions to host cells, in an effort to protect the surrounding tissue or promote repair mechanisms. 

## 5. Current Limitations in the Use of AM and AF Cells

Recent evidence suggests that diverse subpopulations of multipotent cells in amniotic fluid differ in marker expression, morphology, and/or growth kinetics [16, 156]. Furthermore, amniotic membrane-derived cells are not as homogeneous as previously thought. Different culture conditions and methods for isolating and expanding cells with stem cell characteristics might introduce a bias towards producing particular subpopulations of cells [11]. In addition, the gestational stage at which AF is collected [79] and the passage number of the cultured cells [157] will likely influence the resulting cell phenotypes and behaviour. At present, it is not clear exactly what effects these methodological differences have on the outcome of studies, but there is an agreement that cells used by different research groups may not represent identical biological properties. While this renders the comparison of different studies very difficult, it also prompts the question of whether different subpopulations of multipotent cells in AF and AM have distinct differentiation capacities. There is, in fact, some evidence that this is the case [156, 158, 159]. Further exploration of this issue is required, and hopefully it will be possible to exploit these differences to isolate cells with greater potential to differentiate into desired functional cell types. This should be done in conjunction with an examination of the role of culture conditions in directing AF, AE, and AMS cell differentiation towards particular cell fates. 

Furthermore, it is possible that predifferentiation of AF- or AM-derived cells toward a desired phenotype prior to transplantation might promote engraftment in some tissues [160, 161]. This issue warrants further investigation, especially considering the low rate of differentiation of transplanted AM- or AF-derived cells observed in many studies. 

Finally, although AM and AF-derived cells reportedly possess low immunogenicity and can survive transplantation into xenogeneic or allogeneic hosts [14, 20, 61, 62, 146, 162], one study found that AF cells were rejected upon transplantation into immunocompetent animals due to the recruitment of host T and B lymphocytes, natural killer cells and macrophages [163]. In another case, poor survival of amniotic epithelium grafts was observed in mice that received repeated transplants, because of immune rejection [164]. Other studies have also reported a low rate of survival of transplanted AF cells [114, 165, 166], which may be a result of immune rejection. Thus, as for ES cells, whose status as immune-privileged has been questioned [167], further research is required in order to understand the immunological properties of AM- and AF-derived cells, and to enhance graft survival.

**Future Perspectives**

There is a need for the establishment of national and international registries of cell lines derived from amniotic membrane and fluid in order to make these lines available to researchers worldwide. This strategy will facilitate the development of guidelines for the derivation and characterization of new cell lines and provide detailed protocols for culturing and differentiating existing lines. It is expected that the proposed approach would reduce methodological variabilities, which are compounded by the inherent heterogeneity of amniotic cells. In addition, the creation of a library of information pertaining to the research and (pre)clinical use of AF, AE, and AMS cells would allow researchers to choose the most appropriate cell line for a particular application, hopefully leading to more rapid development of effective regenerative therapies.

## Figures and Tables

**Table 1:** Comparison of ES, AM and AF stem cells.

	Embryonic stem cells	Amniotic epithelial cells	Amniotic mesenchymal stromal cells	Amniotic fluid cells
Source	Inner cell mass of preimplantation embryo	Amniotic membrane	Amniotic membrane	Amniotic fluid
*In vitro* lifespan	300+ population doublings [48]	14 population doublings [49]	5–10 passages [50], 27 population doublings [51]	55 [52] to 250+ [14] population doublings
Differentiation potential *in vitro *	Ectodermal, mesodermal, endodermal [53]	Ectodermal, mesodermal, endodermal [20]	Ectodermal, mesodermal, endodermal [20]	Ectodermal, mesodermal, endodermal [14]
Tumorigenicity	Yes [54]	No [15]	Not known	No [14]
Ethical issues	Yes	No	No	No
Clinical trials	Yes [55]	Yes [56]	No	No

**Table 2:** Applications of AF stem cells.

AF cell source	Target tissue	Animal/disease model	Delivery route	References
Human	Brain	Normal and twitcher neonatal mice	Intracerebroventricular injection	[14]
Human	Brain	Mouse cerebral ischemia	Intracerebroventricular injection	[109]
Human	Brain	Rat cerebral ischemia	Intrastriatal injection	[110]
Rat	Brain	Rat cerebral ischemia	Intravenous injection into the jugular vein	[111]
Human	Brain	Mouse motor cortex injury	Injection or implantation of cells seeded on biocompatible scaffolds into the motor cortex	[112]
Human	Nerve	Rat sciatic nerve crush injury	Injection or implantation of cells and fibrin glue into the injury site	[113–117]
Human	Nerve, Muscle	Rat sciatic nerve crush injury	Intravenous injection	[118]
Human	Heart	Rat cardiac infarction	Intracardiac injection of cells or cell sheet fragments	[119, 120]
Rat	Heart	Rat cardiac infarction	Intracardiac injection	[108, 121, 122]
Human	Lung, Heart	Rat pulmonary hypertension and heart failure	Intravenous injection into the tail vein	[123]
Sheep	Heart valve	Fetal sheep	Closed-heart implantation of cells seeded on biodegradable scaffolds *in utero *	[104]
Mouse	Skeletal muscle	Mouse spinal muscular atrophy	Intravenous injection into the tail vein	[124]
Human	Bone	Mouse subcutaneous implantation	Subcutaneous implantation of cells printed on biocompatible polymers	[14]
Rabbit	Bone	Rabbit chest wall/sternal defects	Bone graft implantation of cells seeded on biocompatible scaffolds into the injury site	[99]
Human	Bone	Rat subcutaneous implantation	Subcutaneous implantation of cells seeded on biocompatible polymers	[101]
Sheep	Cartilage	Fetal lamb tracheal reconstruction	Tracheal implantation of cells seeded on biocompatible scaffolds *in utero *	[96]
Sheep	Diaphragm	Postnatal sheep diaphragmatic hernia	Diaphragmatic implantation of cells seeded on biocompatible scaffolds	[97]
Human	Kidney	Mouse kidney acute tubular necrosis	Injection into the renal cortex	[106]
Rat	Bladder	Rat cryo-injured bladder	Intravascular injection	[105]
Rat	Abdomen	Rat	Intraperitoneal injection	[125]
Rabbit	Fetal membranes	Fetal rabbit iatrogenic membrane defect	Injection into the plug followed by fixation to the fetal membranes	[126]
Sheep	Nonspecific	Fetal lamb organs	Injection into the fetal peritoneal cavity *in utero *	[127]
Mouse, Human	Hematopoietic	Mouse	Intravenous injection into the retro-orbital vein	[128]
